# shinyMethyl: interactive quality control of Illumina 450k DNA methylation arrays in R

**DOI:** 10.12688/f1000research.4680.2

**Published:** 2014-09-19

**Authors:** Jean-Philippe Fortin, Elana Fertig, Kasper Hansen

**Affiliations:** 1 Department of Biostatistics, Johns Hopkins Bloomberg School of Public Health, Baltimore, MD, 21205, USA; 2 Department of Oncology, Sidney Kimmel Cancer Center, Johns Hopkins School of Medicine, Baltimore, MD, 21205, USA; 3 McKusick-Nathans Institute of Genetic Medicine, Johns Hopkins School of Medicine, Baltimore, MD, 21205, USA

## Abstract

We present shinyMethyl, a Bioconductor package for interactive quality control of DNA methylation data from Illumina 450k arrays. The package summarizes 450k experiments into small exportable R objects from which an interactive interface is launched. Reactive plots allow fast and intuitive quality control assessment of the samples. In addition, exploration of the phenotypic associations is possible through coloring and principal component analysis. Altogether, the package makes it easy to perform quality assessment of large-scale methylation datasets, such as epigenome-wide association studies or the datasets available through The Cancer Genome Atlas portal. The shinyMethyl package is implemented in R and available via Bioconductor. Its development repository is at https://github.com/jfortin1/shinyMethyl.

## Introduction

The recent release of the R package *shiny* ^1^ has substantially lowered the barriers to interactive visualization in R, opening the door to interactive exploration of high-dimensional genomic data.

DNA methylation is an epigenetic mark, and changes in DNA methylation have been associated with various diseases, such as cancer ^2^. For DNA methylation data, thousands of samples from the state-of-the-art Illumina 450k methylation array ^3^ have been generated and are accessible online from The Cancer Genome Atlas (TCGA) and through the Gene Expression Omnibus (GEO). This array has a series of probes used to measure a methylation and an unmethylation signal for a series of loci. Probes are designed using two main chemistries resulting in a challenging array design, essentially a mix of a two color and a one color array discussed in Bibikova *et al.* ^3^. Analysis of data from this array requires careful quality control and pre-processing that account for these distinct chemistries. The assessment of these steps could benefit from an interactive visualization tool.

Our solution is *shinyMethyl*, an interactive visualization package for 450k arrays, based on the packages *minfi* ^4^ and *shiny* ^1^. The goal of shinyMethyl is two-fold; (1) to help with quality assessment and (2) to help with assessing the effect of pre-processing. We use pre-computation to enable interactive visualization of thousands of samples to circumvent computational bottlenecks during data exploration. The pre-computation can happen on a large computing server and the resulting data object can be used for interactive visualization on a laptop. Quality control and pre-processing large 450k datasets become easy and intuitive with *shinyMethyl*.

## Methods

### shinyMethyl workflow

**Figure 1.**
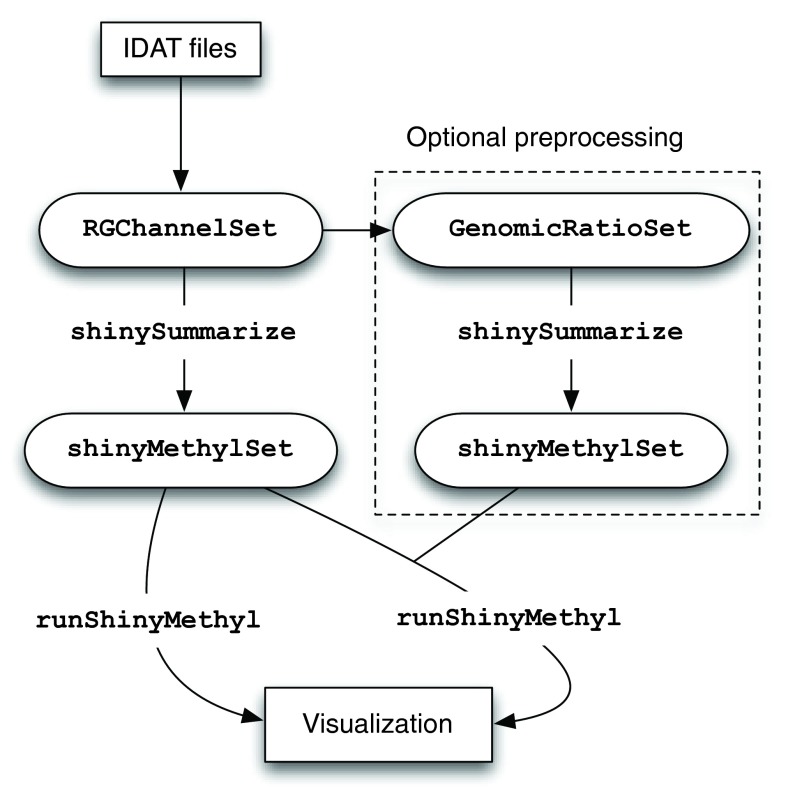
The workflow of *shinyMethyl*. IDAT files are parsed using *minfi* and *illuminaio* into a RGChannelSet. This object is summarized using shinySummarize. Optionally, the data are pre-processed and the pre-processed data are summarized. For visualization, runShinyMethyl is used on either one or two sets of summarized data.

The first step of *shinyMethyl* is pre-computation of various summaries of the 450k array data, using the function shinySummarize. This pre-computation is run on raw (not pre-processed) data and – optionally – pre-processed data, resulting in either one or two summary objects, as described below. These summary objects, called shinyMethylSet, are saved in a platform-independent format. The interactive interface is then launched via the function runShinyMethyl. The function requires a shinyMethylSet containing the summary data from the raw data. In addition, the function accepts as a second argument a shinyMethylSet that contains summaries from pre-processed data, in which case both raw and pre-processed data will be displayed in the interactive interface. Figure 1 illustrates the *shinyMethyl* workflow.

### Raw data summarization

**Figure 2.**
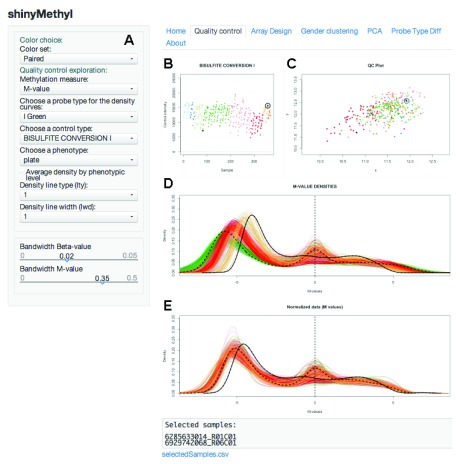
The shinyMethyl user interface for quality control. The interface shows an example interactive visualization of batch effects and quality control (TCGA head and neck squamous cell carcinoma, HNSCC dataset). The interface is divided into a user menu and a plotting area. ( **a**) A menu containing a number of user-settable visualization parameters. The “phenotype” is set to “plate” which makes the color scheme reflect batch. The four plots ( **b**– **e**) are interactive and react simultaneously to the user mouse clicks, so that samples selected on one plot are immediately highlighted on the additional plots. The solid lines in black represents the sample(s) currently selected by the user and match the dot circled in black on ( **b**, **c**). The dashed lines in black represents another sample, previously selected by the user and match the black dot without the circle. ( **b**) Average negative control probes intensities; ( **c**) the median intensity of the M channel against the median intensity of the U channel; ( **d**– **e**) M-value densities for Infinium I probes before and after functional normalization.

Summarizing the raw data uses the *minfi* ^4^ and *illuminaio* ^5^ R packages to parse Illumina IDAT files into a *minfi* object called RGChannelSet. shinySummarize operates on this RGChannelSet and the summarization object created by this function is 35x smaller than the full data representation in *minfi*; 1,000 samples use 205 MB. Specifically, the summarized data contain the quantile distributions of the raw intensities for the unmethylated (U) and methylated (M) channels, copy numbers (CN = M + U), Beta values (Beta) and M values (M-Val). The object contains also the raw control probes intensities and the results of the principal component analysis performed on the autosomal Beta values. The function also extracts the phenotype variables stored in the RGChannelSet. The summarization is done separately by probe types (I and II, see Bibikova *et al.* ^3^) and for sex chromosomes. An S4 class, called shinyMethylSet, is used to represent the data in R, and this object is independent of the operating system. The *shinyMethyl* interface is launched by passing the shinyMethylSet to the function runShinyMethyl. An example of the interface is shown in Figure 2.

### Pre-processed data summarization (optional)

**Figure 3.**
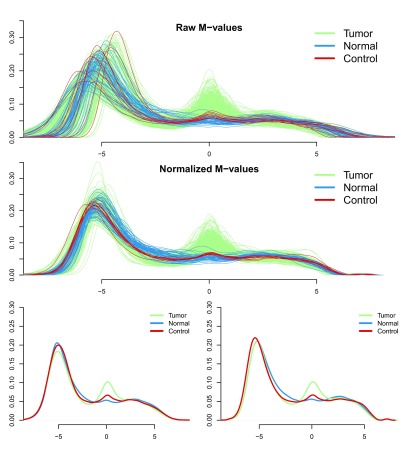
Visualization of cancer/normal differences in the TCGA dataset, before and after normalization. In the first two plots are shown the densities of the M-values for Type I green probes before ( **a**) and after ( **b**) functional normalization as presented in the shinyMethyl interactive interface. Green and blue densities represent tumor and normal samples respectively, and red densities represent 9 technical replicates of a control cell line. The last two plots show the average density for each sample group before and after normalization. Functional normalization preserves the expected marginal differences between normal and cancer, while reducing the variation between the technical controls (red lines).

Summarizing pre-processed data in *shinyMethyl* operates on an S4 object in *minfi* termed GenomicRatioSet. The summaries of the pre-process data are stored in an additional shinyMethylSet. Again, the summarized data object is substantially smaller than the full data representation in *minfi*. If this shinyMethylSet is also included in the runShinyMethyl command, the summaries of the pre-processed data are automatically added to the *shinyMethyl* interface. This option represents a powerful diagnostic tool to assess the global performance of a normalization method, such as plate effect correction ( Figure 2), or preservation of the expected biological differences between different tissues or conditions ( Figure 3).

### Quality control assessment 

Once the DNA methylation data have been summarized, *shinyMethyl* offers three interactive plots for quality control. These plots react conjointly to the user mouse: (1) a density plot of the M/Beta values, (2) a QC plot proposed in *minfi* and (3) a plot of control probes intensities. The samples are colored by a phenotype variable selected by the user. The three plots together allow the user to select aberrant samples, whose array identifiers are saved into a csv file for exclusion in subsequent analyses (outside of *shinyMethyl*). An example of quality control panel is presented in Figure 2 in which summaries from the TCGA head and neck squamous cell carcinoma (HNSCC) samples are colored by batch; *shinyMethyl* allows to observe significant batch effects, a source of obscure variation that has critical consequences in downstream analysis ^6^.

### Sex prediction

The sex of the samples can be accurately predicted by using the intensities of the probes mapping to the sex chromosomes in the M and U channels ^4^. *shinyMethyl* implements this prediction algorithm and allows the user to interactively specify a cutoff to cluster samples by sex.

**Figure 4.**
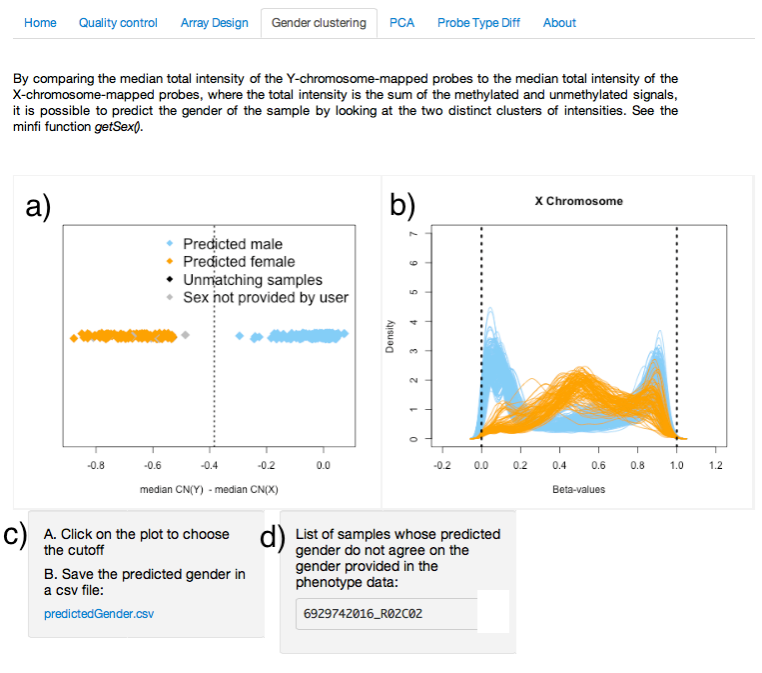
Sex prediction interface. The difference of the median copy number intensity for the Y chromosome and the median copy number intensity for the X chromosome can be used to separate males and females. In **a**), the user can select the vertical cutoff (dashed line) manually with the mouse to separate the two clusters (orange for females, blue for males). Corresponding Beta-value densities appear in **b**) for further validation. The predicted sex can be downloaded in a csv file in **c**), and samples for which the predicted sex differs from the sex provided in the phenotype will appear in **d**).

The array identifiers of the samples for which the predicted sex does not agree with the user-provided sex phenotype are displayed within the interface and can be saved into a csv file for further analysis. From the HNSCC TCGA dataset (described in Example data), one sample shows discrepancy, indicating possible mislabeling ( Figure 4).

### PCA analysis and design confounding

*shinyMethyl* also performs a principal component analysis (PCA) on the 20,000 most variable autosomal probes. This analysis enables the observation of associations between phenotype and methylation levels. An additional panel displays the physical arrays colored by phenotype. This coloring allows the user to discern potential confounding between phenotype and study design.

### Example data

The data package *shinyMethylData* contains the summarized data for 369 HNSCC cancer samples from TCGA. It is available from the Bioconductor project ( http://www.bioconductor.org). All analyses were performed on raw IDAT intensity files available from Level I data in the TCGA Data Portal ( https://tcga-data.nci.nih.gov/tcga). Both raw intensities and normalized methylation values obtained by functional normalization using control probes and a slide covariate ^7^ are included. The shinyMethylSet objects containing respectively the raw and normalized data can be accessed by summary.tcga.raw and summmary.tcga.norm.

## Discussion

*shinyMethyl* makes the quality control and pre-processing of 450k methylation array data fast and intuitive through an interactive application in R. We also show, by example, how to use *shiny* to develop interactive visualization interfaces. Our example will facilitate future developments of interactive visualization tools for the processing of high-dimensional genomic data in subsequent Bioconductor ^8^ packages.

## Software availability

### Software access

*shinyMethyl* is an R package available from the Bioconductor project ( http://www.bioconductor.org). A demo deployment of the software is available at http://spark.rstudio.com/jfortin/shinyMethyl; we caution that this free hosting of the package at times appear much slower than a local installation.

### Latest source code

https://github.com/jfortin1/shinyMethyl

### Source code as at the time of publication

https://github.com/F1000Research/shinyMethyl/releases/tag/v1.0

### Archived source code as at the time of publication

http://dx.doi.org/10.5281/zenodo.10748 ^10^

### Software license

Artistic-2.0
