# Supplementary material for: shinyMethyl: interactive quality control of Illumina 450k DNA methylation arrays in R
Source: F1000Res. 2014 Sep 19;3:175. Originally published 2014 Jul 30. [Version 2] doi: 10.12688/f1000research.4680.2 (PMC4176427; doi:10.12688/f1000research.4680.2)
Supplement: Peer review history

# Peer review: Referee response for version 2

**DOI:** 10.5256/f1000research.5691.r8632

**Published:** 2015-05-12

**Authors:** Timothy Triche

**Affiliations:** 1 Center for Personalized Medicine, University of Southern California, Los Angeles, CA, USA

The authors proved to be correct in that Rstudio has "transitioned" their spark server to a subscription model for shiny hosting. Perhaps my request was unwarranted. The software itself behaves as advertised and is an efficient tool for quality control of these arrays.

I have read this submission. I believe that I have an appropriate level of expertise to confirm that it is of an acceptable scientific standard.

# Peer review: Referee response for version 1

**DOI:** 10.5256/f1000research.5001.r5620

**Published:** 2014-09-03

**Authors:** Tiffany Morris

**Affiliations:** 1 UCL Cancer Institute, University College London, London, UK

This manuscript written by Fortin *et al.* presents an R package to assess the quality control of 450k methylation array data through an interactive interface. A unique aspect of *shinyMethyl* is the use of the shiny package to allow the interactive visualisation. *shinyMethyl* is easy to use and therefore suitable for a novice data analyst with limited programming experience and also experienced bioinformaticians that would like to quickly assess large datasets. Additionally the package utilises the established and widely used R package *minfi* for the data analysis.

*shinyMethyl* is available for download from Bioconductor or github. In this manuscript, the authors clearly describe the use of the package through a workflow and various screenshots and plots. They discuss the example dataset that is available through TCGA. Data interpretation is only explained briefly as the purpose of this manuscript is to present the interface.  In depth background information on analysis methods (terminology, normalisations etc) and details of data interpretation can be found in the *minfi* publication or the Bioconductor vignette.

The package does require the raw IDAT files. Some publicly available datasets may not provide this information and therefore *shinyMethyl *would not be applicable. However, it is becoming mainstream to provide this data and with more packages utilising this raw data it is expected that investigators make it available. 

The authors have clearly presented their package that is well designed, easy to use and a useful addition to the options for 450k data analysis especially as it is the platform of choice for large epigenome-wide association studies.

I have read this submission. I believe that I have an appropriate level of expertise to confirm that it is of an acceptable scientific standard.

# Response

**Published:** 2014-09-15

**Authors:** Kasper Hansen

**Affiliations:** khansen@jhsph.edu, USA

Thank you very much for taking the time to review our work and for the kind referee report.

# Peer review: Referee response for version 1

**DOI:** 10.5256/f1000research.5001.r5619

**Published:** 2014-08-19

**Authors:** Timothy Triche

**Affiliations:** 1 Center for Personalized Medicine, University of Southern California, Los Angeles, CA, USA

The title of the report is entirely appropriate. 

The abstract would benefit from mention of the interactive demo on spark.rstudio.com, as the interface is trivially easy to grasp while performing the various quality control checks.  

The methods not directly treated in this report (functional normalization, Shiny internals, 450k chip design) have been discussed elsewhere at length.The discussion of design and implementation decisions for shinyMethyl is sufficient (again an interactive/exploratory session will provide the interested user with most if not all of the same information). 

The conclusion (shinyMethyl eases 450k quality control for large datasets with potential technical artifacts) is justified in the report, the included example for the package, and the interactive demo hosted by Rstudio.

The data for the example are available as raw binary IDAT files from the TCGA data portal; reproducibility via minfi is trivial, provided the user has sufficient compute resources to process and normalize a large dataset. (In my experience, datasets of 1000+ samples can be read with illuminaio and normalized via funnorm() on a typical Linux server with 48-64GB of RAM) 

This report serves a useful purpose (beyond the announcement of the software) by providing a usable baseline for 450k quality control. Epigenome-wide association studies are prone to a staggering number of potential confounding factors, not least of which are technical and experimental design artifacts. Investigators who have an interest in reproducible conduct of such studies now have further incentive to use and deposit raw binary IDAT files for their experiments. Investigators who choose not to do so may be viewed with skepticism, especially when the data has been processed for analysis via minfi (or, for that matter, methylumi), and verification of straightforward quality controls is made simpler by the interactive shinyMethyl package.

I have read this submission. I believe that I have an appropriate level of expertise to confirm that it is of an acceptable scientific standard.

# Response

**Published:** 2014-09-15

**Authors:** Kasper Hansen

**Affiliations:** khansen@jhsph.edu, USA

We thank the referee for taking the time to review our work, and thank for the kind review.

We have submitted a revision with an added sentence about the demo at spark.rstudio.com under "Software access", but caution that this website at times is slow to respond to input. This is a hosting service freely available to the community.
